# The Evolving Treatment Landscape for the Elderly Multiple Myeloma Patient: From Quad Regimens to T-Cell Engagers and CAR-T

**DOI:** 10.3390/cancers17152579

**Published:** 2025-08-05

**Authors:** Matthew James Rees, Hang Quach

**Affiliations:** 1 Department of Clinical Haematology, St Vincent’s Hospital Melbourne, Fitzroy, VIC 3065, Australia; 2 Division of Hematology, Mayo Clinic, Rochester, MN 55905, USA; 3 Department of Medicine, University of Melbourne, Parkville, VIC 3010, Australia

**Keywords:** elderly, multiple myeloma, CAR-T therapy, T cell engagers, immunomodulatory agents, proteasome inhibitors

## Abstract

Multiple myeloma (MM) is predominantly a disease of the elderly. In recent years, a surge of highly effective plasma cell therapies has revolutionized the care of elderly multiple myeloma (MM) patients, for whom frailty and age-related competing causes of mortality determine management. Traditionally, the treatment of newly diagnosed elderly patients has centered on doublet or triplet combinations composed of immunomodulators (IMIDs), proteasome inhibitors (PIs), anti-CD38 monoclonal antibodies (mAbs), and corticosteroids producing median progression-free survival (PFS) rates between 34 and 62 months. However, recently, a series of large phase III clinical trials examining quadruplet regimens of PIs, IMIDs, corticosteroids, and anti-CD38 mAbs have shown exceptional outcomes, with median PFS exceeding 60 months, albeit with higher rates of peripheral neuropathy (≥Grade 2: 27% vs. 10%) when PIs and IMIDs are combined, and infections (≥Grade 3: 40% vs. 29–41%) with the addition of anti-CD38mAbs. The development of T-cell redirecting therapies including T-cell engagers (TCEs) and CAR-T cells has further expanded the therapeutic arsenal. TCEs have shown exceptional activity in relapsed disease and are being explored in the newly diagnosed setting with promising early results. However, concerns remain regarding the logistical challenges of step-up dosing, which often necessitates inpatient admission, the infectious risks, and the financial burden associated with TCEs in elderly patients. CAR-T, the most potent commercially available therapy for MM, offers the potential of a ‘*one and done*’ approach. However, its application to elderly patients has been tempered by significant concerns of cytokine release syndrome, early and delayed neurological toxicity, and its overall tolerability in frail patients. Robust data in frail patients are still needed. How CAR-T and TCEs will be sequenced among the growing therapeutic armamentarium for elderly MM patients remains to be determined. This review explores the safety, efficacy, cost, and logistical barriers associated with the above treatments in elderly MM patients.

## Simple Summary

Elderly individuals account for approximately one-third of multiple myeloma (MM) diagnoses, yet they also represent a highly heterogeneous population due to medical comorbidities, frailty, and social factors. There have been dramatic therapeutic advances in MM—including the establishment of triplet and quadruplet regimens containing anti-CD38 monoclonal antibodies in newly diagnosed MM, bispecific T-cell engagers, and chimeric antigen receptor (CAR) T-cell therapies. Elderly and frail individuals were underrepresented in the pivotal clinical trials which defined these therapies as standard of care. Nonetheless, the distinct, non-chemotherapeutic toxicity profile of these agents makes them particularly well suited for elderly patients. As such, immunotherapies hold the potential to improve outcomes in this vulnerable group, but their adoption has been hampered by concerns about tolerability, access, logistical complexity, and cost. Herein, we review evidence on the safety, efficacy, and barriers to adopting these therapies in elderly MM patients.

## 1. Introduction

Multiple myeloma (MM) is primarily a disease of the elderly, with a median age at diagnosis of 69 years; one-third of newly diagnosed patients are over 75 years and 10% are over 85 years of age [1]. With the profound shift in the age of the world’s population, the number of MM patients aged ≥ 65 years is expected to rise by more than 36% between 2020 and 2030 [2]. Despite their growing prevalence, elderly MM patients are underrepresented in clinical trials and their management is largely extrapolated from clinical trials of transplant-ineligible patients, leading to a significant research gap [3,4]. This is relevant because elderly patients are a heterogenous group subject to the effects of frailty, medical comorbidities, and increased susceptibility to adverse treatment effects [3]. Consequently, treatment strategies for elderly patients must be appropriately tailored, balancing efficacy with quality of life, and aiming to maximize efficacy while preserving function and independence.

Over the past 20 years we have seen an improvement in survival outcomes for older MM patients, driven primarily by the development of immunomodulatory drugs (IMIDs), proteasome inhibitors (PIs), and CD38-directed monoclonal antibodies (mAbs). In the early 2000s, patients ≥ 65 years had a median OS of only 4.6 years, in comparison to modern series where patients ≥ 70 years can expect a median OS beyond 6.5 years [5,6,7]. Notably, elderly patients have derived less of a survival benefit over the past two decades compared to the general MM population, and this difference is not clearly explained by differences in age and high-risk disease features [8]. While some high-risk cytogenetic features are more common in the elderly, their prognostic impact appears to diminish with age, while the impact of patient-related factors is enhanced [9,10]. The reduced benefit of therapeutic advances highlights the need for improved approaches for elderly MM patients, especially as the treatment landscape experiences an unprecedented upheaval following the introduction of T-cell engagers (TCEs) and chimeric antigen receptor T-cells (CAR-T).

The application of immunotherapies to elderly MM patients is a key clinical question and this review will examine modern approaches for newly diagnosed elderly MM patients, focusing on emerging immunotherapeutic strategies. For the purposes of this review we will consider elderly MM patients as those aged ≥ 75 years, as such patients are almost unanimously considered ineligible for intensive therapeutic approaches such as front-line autologous stem-cell transplant (ASCT) [11].

## 2. General Considerations in the Treatment of Elderly Myeloma Patients

Frailty is a state of decreased reserve affecting multiple organ systems and is a key determinant of treatment delivery in elderly MM patients [12]. As a result, several clinical tools have been developed to assess frailty in MM, including the IMWG frailty score, the revised myeloma comorbidity index, the Mayo risk score, and the IFM simplified frailty assessment [13,14,15,16,17]. While such tools have been shown to consistently predict survival, and treatment discontinuation in MM, their adoption in routine clinical practice is limited, primarily due to time constraints. Another limitation of these tools is the omission of physical performance tests, including gait speed, and grip strength. We note that a reduction in gait speed has been associated with increased mortality and a high frequency of hospitalizations in MM [18]. Irrespective of frailty, age is independently associated with survival and there is a stepwise reduction in PFS and OS with each additional decade of life [19]. However, age alone should not be considered a marker of frailty, and this remains a limitation of all the aforementioned scores, which include age as a parameter of frailty. Age by itself does not cause frailty [13,14,15,16].

In addition to existing clinical frailty scores, there is growing interest in incorporating objective biomarkers of frailty, such as sarcopenia, into these frameworks. Sarcopenia refers to the loss of skeletal muscle mass and function, and is associated with increased treatment-related toxicity and reduced tolerance to therapy and inferior survival [20,21,22]. Moreover, frailty should be viewed as a dynamic and potentially reversible condition, particularly in the context of clinical improvement or toxicity-related deconditioning [14,15]. Future efforts should prioritize frailty-informed trial designs and tools that can capture real-time changes in patient fitness to better individualize therapy for elderly patients with myeloma [23].

The approach to elderly and frail patients with MM remains a challenge due to underrepresentation in pivotal clinical trials, a lack of high-quality prospective trials, and absence of standardized treatment recommendations. Although clinical trial design is evolving to incorporate a frailty- or fitness-based approach, many of the contemporary treatment strategies discussed in this review rely on the traditional distinction between transplant-eligible and transplant-ineligible patients [24].

Several trials have explored frailty-adapted treatment approaches in multiple myeloma. The RV-MM-PI-0752 study compared fixed-duration Rd-R versus continuous Rd in frail patients and demonstrated improved tolerability and outcomes with the fixed-duration approach [25]. The IFM 2017 trial assessed treatment strategies in frail patients and supported the feasibility of dexamethasone-sparing regimens [26]. Lastly, the UK FITNESS trial is evaluating Ixazomib-Rd with or without frailty-guided dose modifications, aiming to optimize treatment delivery based on patient fitness [27].

## 3. Triplet and Quadruplet Therapy

Historically, transplant-ineligible newly diagnosed MM has been managed with either lenalidomide–dexamethasone (Rd) or an alkylator-based doublet (melphalan-prednisone, MP) or triplet (melphalan-prednisone-thalidomide/bortezomib, MPT, MPV) combination [28,29,30], with the former being the preferred regimen in the USA, and the latter often used in Europe. As both PFS and OS were shown to favor Rd over MPT among patients aged ≥ 75 years in the FIRST trial, this became the standard of care regimen in elderly MM patients [29,31].

The benefit of adding daratumumab (D), a monoclonal anti-CD38 directed antibody, to the above regimens was confirmed in two large phase III trials; MAIA (DRd vs. Rd) and ALCYONE (D-VMP vs. VPM), Table 1. ALCYONE demonstrated that the addition of daratumumab substantially deepened disease responses (CR rate: 43% vs. 24%, MRD negativity 10^−5^: 22% vs. 6%), and that elderly patients ≥ 75 years experienced superior OS, although this did not reach statistical significance [32,33]. Despite these impressive results, the reduced secondary leukemogenicity, incidence of myelosuppression, and neuropathy with Rd compared to VMP means that VMP is no longer a standard-of-care for MM.

It was the MAIA trial which had the most profound impact on contemporary practice: here Rd was compared to DRd until intolerance or disease progression in a cohort of truly transplant-ineligible patients (median age 74 years), producing unprecedented survival outcomes, with median PFS eclipsing 5 years for patients who received DRd [7,34]. Like ALCYONE, deeper CR and MRD negativity rates were observed, and the signal for superior PFS (HR = 0.59, 95%CI = 0.44–0.79) and OS (HR = 0.75, 95%CI 0.55–1.02) was even stronger among patients ≥ 75 years [7,34]. Notably, in both ALCYONE and MAIA, the addition of daratumumab was associated with higher rates of infection, in particularly pneumonia (G3 + ALCYONE: 11% vs. 4%, MAIA: 19% vs. 11%) [7,32,33,34]. An important insight from MAIA concerns the dose intensity of lenalidomide, which is often poorly tolerated among older individuals due to gastrointestinal effects and fatigue. Patients treated with DRd received a lower median dose intensity of lenalidomide and more frequently discontinued lenalidomide (37% vs. 24%) compared to the Rd arm, but despite this they still experienced superior PFS and OS—implying that the use of this triplet combination permits lower but still effective doses of lenalidomide to be used [7,34].

In 2024 three major phase III clinical trials were published, challenging the dominance of the DRd regimen: these were the CEPHEUS, IMROZ and the BENEFIT trials [37,38,39]. All trials investigated a quadruplet regimen containing an anti-CD38 mAb, with the proteasome inhibitors bortezomib, lenalidomide, and dexamethasone (VRd) as the experimental arm. The motivation for the addition of an anti-CD38 mAb to a VRd background in transplant-ineligible MM stems from the SWOG777 study (VRd vs. Rd). While SWOG777 was not a transplant ineligible trial or a trial designed for elderly patients, it nonetheless demonstrated superior OS and PFS with VRd compared to Rd among patients aged ≥ 65 years and so became a standard of care for transplant ineligible patients [35,36]. A major shortcoming of SWOG777 was the use of intravenous bortezomib administered twice weekly, contributing to a higher rate of neuropathy and treatment discontinuation and this has now replaced with subcutaneous dosing [40]. In the case of all three quadruplet trials, the efficacy of the quadruplet was superior to the triplet comparator [37,38,39]. In IMROZ and CEPHEUS (anti-CD38-VRd vs. VRd alone), the MRD negativity rate was significantly higher with the mAb (CEPHEUS: 61% vs. 39%, IMROZ: 58% vs. 44%) and both met their PFS primary endpoints [37,38]. In IMROZ, the 5-year PFS was 63% with isatuximab-VRD (Isa-VRd) compared to 45% with VRd, despite an exceptional performance in the VRd arm.

In contrast to CEPHEUS and IMROZ which examined the benefit of adding an anti-CD38mAb to VRd, the BENEFIT trial (Isa-Rd vs. Isa-VRd) set out to answer the utility of adding a proteasome inhibitor to an Isa-Rd backbone akin to MAIA. BENEFIT confirmed that the addition of bortezomib significantly improved the MRD 10^−5^ negativity rate at 18 months from randomization (26% vs. 53%), as well as all other MRD endpoints from 12 months [39]. The most pertinent finding of BENEFIT was an estimate of the ‘*cost*’ of adding bortezomib when treating elderly MM patients, as assessed through the incidence of peripheral neuropathy (Isa-VRd, any grade: 52%, Grade ≥ 2: 27% vs. Isa-Rd, any grade: 28%, Grade ≥ 2: 10%). While the MRD results are attractive, over one quarter of patients experienced clinically significant peripheral neuropathy with the administration of weekly subcutaneous bortezomib for three out of four weeks, and so this must be evaluated with caution in elderly patients [39].

While all three trials were conducted at an exceptional standard, few patients ≥ 75 years were included and no patients ≥ 80 years were included (19% of MAIA population aged ≥ 80 years), limiting the extrapolation of these results to elderly patients [34,37,38,39]. Regarding frailty, both CEPHEUS and BENEFIT trials excluded frail patients (frailty score <2 per the Myeloma Geriatric Assessment score) [37,39]. In contrast, IMROZ permitted the inclusion of frail patients, and 27% of patients were classified as frail using the simplified IMWG frailty score [41]. IMROZ confirmed that even frail patients derived significantly improved PFS with Isa-VRd compared to VRd (HR = 0.52, 95%CI 0.42–0.90, *p* = 0.023) [41]. While this is commendable, it is a still significantly lower representation of frail individuals than the MAIA study, in which nearly half of included patients were categorized as frail using the IFM simplified frailty assessment [15,42]. MAIA confirmed that frail patients fared poorly compared to their non-frail counterparts; however, irrespective of frailty status, daratumumab maintained a significant benefit over Rd alone [42]. In summary, while evidence supports the addition of daratumumab to standard regimens in frail patients, the benefit of incorporating bortezomib in frail patients remains a matter for debate. While bortezomib enhances disease-control, the associated risk of bortezomib-associated peripheral neuropathy, which negatively impacts quality of life and functional independence, needs to be considered carefully.

While carfilzomib (K) appears more effective for high-risk MM [43], it is associated with higher cardiovascular and renal toxicities [44], and so there has been less interest in carfilzomib-based quadruplets in elderly multiple myeloma patients. Two phase 2 trials of an anti-CD38-KRd quadruplet (MANHATTAN and GMMG-CONCEPT) found high rates of MRD negativity 10^−5^ around 70%; however, both trials had limited numbers of patients ≥ 75 years. Furthermore, in the GMMG-CONCEPT trial the transplant-ineligible cohort had 20% cardiac grade ≥ 3 adverse events, underscoring the risk of this approach in elderly patients [45,46].

## 4. Immunotherapies

### 4.1. B-Cell Maturation Antigen

So far, the principal antigenic targets of immunotherapeutic approaches have been the B-cell maturation antigen (BCMA), G-protein coupled receptor, family C, group 5, member D (GPRC5D), and CD38. BCMA is a transmembrane receptor and member of the tumor necrosis factor superfamily [47]. Several characteristics of BCMA make it an ideal immunotherapeutic target. First, BCMA expression is restricted to plasma cells and terminally differentiated B cells, with higher expression on MM cells compared to normal plasma cells [48]. Second, BCMA binding activates downstream signaling via mitogen-activated protein kinase (MAPK) and nuclear factor kappa-B (NFKB) pathways that are essential to plasma cell survival [49,50]. Third, membrane bound BCMA may be released from the cell surface by gamma-secretase mediated shedding to produce soluble BCMA (sBCMA), which has emerging roles as a biomarker for disease response, a surrogate for tumor burden, and as an adjunctive therapeutic target through gamma secretase inhibitors [51,52,53].

### 4.2. G-Protein Coupled Receptor, Family C, Group 5, Member D

GPRC5D is expressed on keratinized tissues such as hair follicles, nail beds, and sweat glands, in addition to malignant plasma cells [54,55]. Due to this expression pattern, patients experience a unique set of off-tumor, on-target adverse effects including skin desquamation, nail changes, xerostomia, and dysgeusia [56]. Recently, cerebellar toxicities have also been reported with both talquetamab and GPRC5d-directed CAR-T potentially due to low-level GPRC5d expression in the cerebellum or inferior olivary nucleus [57,58].

## 5. T-Cell Engagers

### 5.1. T-Cell Engager Design

**Figure 1:**
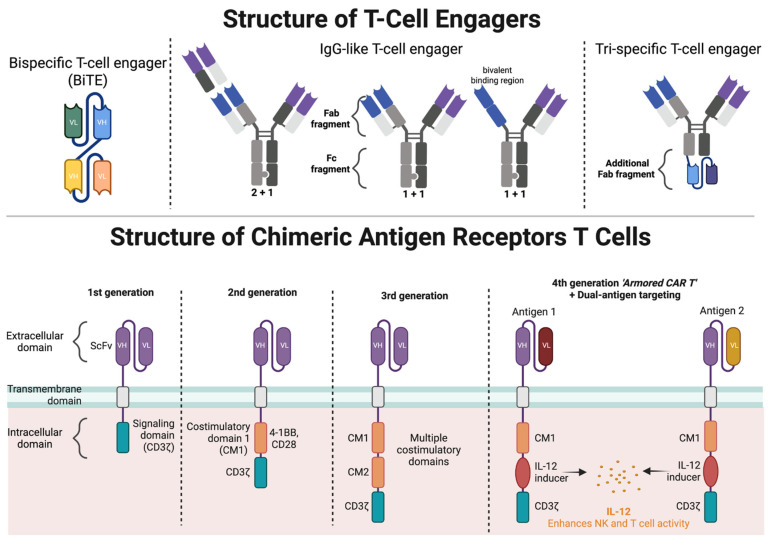
T-cell engager and CAR-T design and structure: the past, present, and future.

TCEs can bind two or more distinct antigens to simultaneously engage T-cells and tumor cells. This dual binding facilitates an immunological synapse between T-cells and tumors cells [59], producing T-cell activation independent of major histocompatibility complex (MHC) restriction and without costimulatory signaling [59,60,61]. TCEs are generally synthesized as recombinant proteins [62], and their design has evolved significantly since the original bispecific T-cell engagers (BiTEs), Figure 1 [59]. Second generation TCEs are characterized by lower affinity CD3 binding to avoid excess T-cell activation and CRS and continued use of Fc-silencing to extend half-life permitting less frequent dosing interval (bi-weekly or monthly). In addition to optimizing pharmacokinetics, further modification of the Fc region can reduce antibody-dependent cellular mediated or complement mediated cytotoxicity, to increase anti-tumor specific T-cell activation [63].

### 5.2. FDA-Approved Bispecific Antibodies

An extensive discussion of all investigational TCEs is beyond the scope of this review; instead, we will focus on FDA-approved TCEs and agents approaching registration. Table 2 summarizes the results of key trials for FDA-approved immunotherapies in relapsed myeloma. Teclistamab and elranatamab are both BCMA-directed TCEs with ORRs of ~60% in heavily pretreated patients; of note, the majority of responses are deep, with ~40% of patients obtaining a CR and the median duration of response for both agents being in excess of 18 months [64,65]. Linvoseltamab, another BCMAxCD3 bispecific antibody, received conditional EMA approval in 2025 for relapsed MM and is currently under evaluation by other regulatory agencies. Talquetamab is the only FDA-approved GPRC5D-directed TCE. Although its efficacy is comparable to teclistamab and elranatamab, its side-effect profile is distinctly secondary to on-target off-tumor effects on keratinized tissues and includes skin, nail, and taste-related changes [56]. While skin and nail changes tend to improve with time, dysgeusia persists and often requires dose reduction in the setting of significant weight loss [56]. The time to response is rapid for all TCEs around 1–2 months [56,64,65]. While the top-line results of the TCE trials are highly promising, frail patients were poorly represented. In MajesTEC-1, MagnetisMM-3, and MontumenTAL-1, only 15%, 23%, and 19% of participants were ≥75 years [56,64,65].

Recently, real-world experience suggested similar efficacy when TCEs are applied to elderly patients, with no difference in ORR, PFS, or OS with increasing age [66,67]. In a multicenter U.S. cohort of 83 patients aged ≥ 75 years treated with teclistamab across 13 academic centers, outcomes mirrored those seen in the pivotal MajesTEC-1 trial, with an ORR of 62% and a median PFS of 10.7 months [68]. Similarly, a multicenter French real-world series of 101 patients receiving elranatamab (median age 68) reported response rates comparable to those observed in the MagnetisMM-3 study, with a manageable safety profile in older adults [69].

**Table 2:** Registrational clinical trials of FDA-approved T-cell engagers and CAR-T in relapsed multiple myeloma.

Agent	Sample Size, *n*=	Median Age Age ≥ 75 Years	HRCA *, %	Median No. Prior Lines	ORR	CR	MRD-Negativity (10^−5^) ^+^	Median Follow-Up (Months)	Median Overall Survival (Months)	Median Progression-Free Survival (Months)	Treatment Discontinuation Due to AEs, %	CRS/ICANS All Grades/Grade ≥ 3	Infections, % All Grades/Grade ≥ 3
**Teclistamab [65,70]**	165	64 15%	26	5	63	46	29	30	22	11	5	CRS: 72/1 ICANS: 3/0	79/55
**Elranatamab [64,71]**	123	68 19%	25	5	61	35	24	28	25	17	14	CRS: 56/0 ICANS: 4/0	70/40
Talquetamab * [56,58]	232	65 23%	16	6	69	40	NA	19	Not reached	11	9	CRS: 80/0 ICANS: 1/0	76/20
**Cilta-cel [72,73]**	97 (113) ^†^	61 8%	24	6	97	67	55	61	61	35	-	CRS: 95/4 ICANS: 17/2	58/20
**Ide-cel [74] ^^^**	128 (140) ^†^	61 8%	35	6	81	39	26	13	19	12	-	CRS: 84/5 Neurotoxicity ^‡^: 18/3	70/NA

* HRCA = t (4;14), t (14;16), and Del17p, + MRD negativity rate defined as the proportion of ITT population obtaining MRD negativity at 10^–5^. * Talquetamab 0.8 mg/kg Q2 weekly schedule. † 113 patients enrolled and apheresed for cilta-cel and 97 patients received cilta-cel infusion; percentages displayed as proportion of patients who received cilta-cel infusion. A total of 140 patients enrolled and apheresed for ide-cel and 128 patients received ide-cel infusion; percentages displayed as proportion of patients who received ide-cel infusion. ^ Response and survival data for 450 × 10^6^ cell dose. ‡ Investigator-identified neurotoxicity was the preferred term. NA, not available.

All TCEs are associated with cytokine release syndrome (CRS), which occurs in 60–70% of individuals and is typically low grade (Grade 1–2). Real-world data of elderly and frail patients confirm TCE-associated CRS is manageable even among these vulnerable patient populations [67,75]. The greatest concern of TCEs in elderly patients stems from the high incidence of ≥grade 3 infections, which exceeds 40% with BCMA-directed TCEs [56,64,65]. Strategies to mitigate infectious complications include adjusted dosing strategies (time-limited vs. response-adapted), appropriate anti-microbial prophylaxis, and immunoglobulin replacement [59,76,77,78].

### 5.3. Trispecific-Antibodies

As design and manufacturing processes evolve, trispecific compounds with two high affinity domains to bind tumor antigens and a single lower affinity domain to CD3 are being investigated [79,80]. Low-affinity CD3 binding appears a promising approach to mitigate CRS and the risk of T-cell exhaustion [81]. Additionally, by possessing antigen binding domains for two plasma cell-specific antigens, the incidence of on-target, off-tumor effects (such as keratinized tissues for GPRC5d) appears to be reduced [80]. Notable trispecific compounds include ISB 2001 a CD3xCD38xBCMA-directed trispecific antibody, whose initial phase 1 results obtained an ORR of 83% at its target dose level, despite high rates of prior BCMA-directed CAR-T and TCE exposure [82]. Equally impressive are the recently presented results of JNJ-79635322 a CD3xBCMAxGPRC5d, which obtained an ORR of 100% at the recommended phase 2 dose (RP2D) in patients naïve to BCMA or GPRC5d directed therapy [80].

## 6. CAR-T Therapy

### 6.1. CAR-T Design and Production

Since their development, there have been several iterations of CAR-T design, Figure 1. At the most fundamental level, the chimeric antigen receptor is composed of an extracellular domain, containing a single-chain variable fragment (scFv) connected to a transmembrane domain and an intracellular domain [83]. Nearly all CAR-T utilize a CD3zeta intracellular stimulatory domain to initiate T-cell activation. Successive generation of CARs have incorporated additional intracellular components to enhance persistence, expansion, and tumor-cell killing, including costimulatory domains (CD28 or 4-1BB) and immunomodulatory molecules (IL-7, CCL19) [83]. Dual-targeting CAR, which express >1 chimeric antigen receptors, appears to provide enhanced MM-cell killing and prevent antigenic escape [84]. Lastly, innovations of CAR-T production including automated closed-system bioreactors, such as the CliniMACS prodigy and Lonza Cocoon, have been developed to reduce production costs and timelines while maintaining product quality [85,86]. Non-viral gene transfer methods, including transposons and CRISPR genome editing, have also been employed to engineer T cells more efficiently and safely [85,86].

### 6.2. FDA-Approved CAR-T

The introduction of CAR-T therapy to relapsed myeloma led to unprecedented results in extensively treated patients, Table 2. While the uptake of CAR-T in elderly patients has been tempered by concerns for excessive toxicity in the form of CRS, immune effector cell-associated neurotoxicity syndrome (ICANS), and delayed neurological toxicity, they remain highly effective in this population. Recently, long-term follow-up from CARTITUDE-1 demonstrated that one-third of patients who were infused with cilta-cel remain alive and progression-free at ≥5 years since treatment [73]. A subset of these patients additionally had serial MRD and PET-CT assessment and all remain MRD-negative at year 5 or later following cilta-cel [73]. These unmatched results suggest that cilta-cel is potentially curative in relapsed MM, or at the very least a means of functional cure for elderly patients. However, both the CARTITUDE-1 and KarMMa-1 studies included limited numbers of elderly individuals (<10%). An older patient subgroup analysis of the KarMMa study demonstrated that treatment outcomes were similar in patients aged ≥ 70 years and those that were younger [87].

Despite the growing role of CAR-T cell therapies in relapsed MM, data specific to elderly and frail patients are limited. A single-center real-world series demonstrated patients ≥ 70 years vs. <70 years had no difference in OS, PFS, or CRS incidence (79% vs. 86%, Grade ≥ 2: 2% vs. 5%) [88]. But, they did experience more ICANS (13% vs. 21%) and infections (all grades; 18% vs. 29%), which was associated with higher healthcare utilization through ED visitations and unplanned hospitalizations [88]. Retrospective real-world series have reported encouraging outcomes in older adults, but also a potentially increased risk of prolonged cytopenias, infections, and delayed functional recovery [89]. Frailty-specific data remain scarce, underscoring the need for prospective studies incorporating geriatric assessments to better understand tolerability and long-term outcomes of CAR-T in this population.

Emerging predictive models for CAR-T-related toxicity may assist in patient selection and risk stratification. Biomarkers such as albumin, CRP, and ferritin, as well as sBCMA and tumor volume all influence CAR-T outcomes in MM [90,91,92]. Their application may be particularly valuable in elderly or borderline-fit patients, where careful risk–benefit assessment is essential.

## 7. Strengths and Weaknesses of T-Cell Redirecting Therapies

Due to the co-emergence of CAR-T and TCEs into the field of relapsed MM, their comparative strengths and weaknesses deserve special mention. Key characteristics of FDA-approved cellular therapies and TCEs, including their weaknesses and strengths are summarized in Table 3. Undoubtedly, cilta-cel has demonstrated the most impressive single agent efficacy to date, the seminal CARTITUDE-1 demonstrating an ORR of 98% median PFS of 34.9 months [73]. However, this efficacy is associated with increased immediate toxicity compared to TCEs, with higher rates and more severe CRS and ICANS than TCEs [56,65,74,93]. Although the time frame of these toxicities is predictable and risk factors for their occurrence (i.e., tumor burden) and strategies for their management have all improved with increasing physician familiarization, their management in frail patients remains a concern [94]. Idiosyncratic toxicities more frequent with certain CAR-T products such as cilta-cel have also been reported, including the delayed onset of neurocognitive events (tremors, psychomotor retardation, inattention, micrographia) [95,96]. Lastly, the potential for fatal complication like immune effector cell-associated hemophagocytic lymphohistiocytosis-like syndrome (IEC-HS) is troubling, particularly in medically frail individuals [97,98].

CAR-T is also limited by manufacturing constraints (including production failures and delays) and a limited number of accredited centers; however, with greater experience and streamlined production processes, the detriment of these factors has been lessened [99]. For instance, in the KarMMa-3 and CARTITUDE-4 studies, 10% and 15% of patients did not receive CAR-T infusion due to disease progression prior to produce availability [95,100]. Improved manufacturing processes, in vivo CAR-T products and allogeneic products may also reduce the period between apheresis and product administration, reducing the need for bridging therapies. The other major strength of CAR-T is the potential for time-off therapy, the so-called ‘*one-and-done*’ approach. While the introduction of post CAR-T maintenance strategies threatens this situation [101], having time free from treatment is associated with improved quality of life, as well as physical and psychological benefits [102]. While the potential for time-limited therapy is being explored with TCEs, this currently remains investigational [103].

By comparison, TCEs offer a highly effective, ‘*off-the-shelf*’ therapy, fulfilling a critical role for patients with a rapid tempo of relapse. CRS and neurological toxicities are less frequent and severe, so much so, that many institutions have implemented or are in the process of adopting an outpatient step-up schedule, obviating the need for inpatient admission. The reduced upfront toxicities also make this therapeutic class more appealing for elderly and frail patients, with no perceived ‘*upper-age threshold*’ to their administration. But undoubtedly the single greatest advantage of TCEs is their readiness to pair with other conventional or investigational TCEs to augment efficacy and permit a reduction in TCE dosing longer term [104,105,106]. The redirecTT study, which investigated the combination of talquetamab and teclistamab in patients with relapsed MM, obtained an ORR of 80% in a cohort with true extra-medullary disease (EMD); moreover, responses were sustained with 86% of patients remaining in response at 18 months [104]. Similarly, the TRIMM-2 study which evaluated the combination of talquetamab and daratumumab in relapsed MM obtained a similar ORR of 78% with median PFS of 19.4 months [106].

In contrast to CAR-T, whose risk profile is high in the period immediately following infusion and which then gradually diminishes, the adverse effect profile of TCEs persists with their ongoing administration. The infectious risk associated with BCMA-directed TCEs, as well as dysgeusia and weight loss associated with GPRC5d-directed therapies, remains troublesome for many patients [58,65,107]. Another important consideration physicians must contemplate is the ideal sequencing of these therapies in MM. Prior BCMA-directed therapy exposure negatively impacts both TCE and CAR-T efficacy, but the magnitude of effect is most pronounced for CAR-T, suggesting CAR-T should be used first when it is being considered [66,107,108,109,110,111].

## 8. Moving Immunotherapy into the Front Line

Treatment attrition, that is the fallout of patients prior to subsequent lines of therapy is a major issue for elderly MM patients. Data from Europe and the USA indicated that less than half of newly diagnosed MM patients will receive a third line of therapy [112,113]. And this attrition rate is likely to be even higher among elderly patients for whom competing causes of mortality are more prominent. In light of this, there is a strong imperative to utilize the most-effective treatment options upfront in elderly MM patients. A summary of phase III trials investigating T-cell redirecting therapy in transplant ineligible MM is shown, Table 4 [3,78]. While the results of these trials are eagerly anticipated, early data from TCEs in newly diagnosed MM appear extremely promising. MajesTEC-5 examined DRd and DVRd in combination with teclistamab in transplant-eligible newly diagnosed MM; here, 100% of participants had obtained MRD negativity (10^−5^ threshold) following only three cycles of treatment [114]. Likewise, MagnetisMM-6, which is examining elranatamab in combination with lenalidomide and daratumumab in transplant-ineligible newly diagnosed MM, has an ORR of 92% with 81% of patients, reaching a very good partial response or better [115].

## 9. Future Directions

With the results of the IMROZ, CEPHEUS, and BENEFIT trials, quadruplet therapy is the new standard of care for newly diagnosed transplant ineligible MM, with DRd or Isa-Rd remaining preferred options for frail individuals [37,38,39]. However, the introduction of TCEs and their rapid transition to the newly diagnosed setting means this current standard of care may not endure for long [115,119]. It remains to be seen whether an upper age limit exists for the administration of CAR-T or TCEs in MM, or whether their toxicity profile will continue to improve with increased familiarization. Mitigating infectious complications appears to be the greatest challenge to the introduction of TCEs in the newly diagnosed setting and may be overcome with fixed-duration therapy or an MRD-guided approach.

Importantly, for many elderly patients, especially those with favorable cytogenetic features and indolent disease biology, TCEs and CAR-T offer the possibility of an ‘operational cure’ with one or two lines of therapy. In this setting, a continuous suppressive therapeutic approach leads to unnecessary toxicity, increases healthcare burden, increases cost, and reduces quality of life. Consequently time-limited or response-adapted approaches for elderly and frail patients may mitigate toxicity, improve treatment adherence, and enhance patient satisfaction.

Besides upfront-combination therapy with TCEs and CAR-T, another potent combination treatment is with the cereblon E3 ligase modulators (CELMoDs), iberdomide, and mezigdomide. Mezigdomide has demonstrated immune-stimulator effects in preclinical studies via enhanced T-cell and NK cell activity, which would synergize with an immunotherapeutic approach [120]. Additionally, CELMoDs have been shown to activate specific T-cell subsets, such as Vγ9Vδ2 T cells, which are crucial for anti-tumor immunity and which have the capacity to reverse immune exhaustion [121]. Other logistic considerations for future research include outpatient step-up dosing for TCEs, and whether the prophylactic use of interventions to reduce CRS can facilitate this including tocilizumab [122].

## 10. Conclusions

The introduction of anti-CD38 monoclonal antibodies, TCEs, and CAR-T therapy has ushered in a transformative era for elderly patients with multiple myeloma. With effective triplet and quadruplet approaches, many elderly MM patients can expect an ‘*operational cure’* with one to two lines of therapy. As TCEs and CAR-T are moved earlier in the treatment algorithm, it is conceivable that select patients will only need a single line of treatment. However, as efficacy improves, minimizing treatment-related toxicities is paramount. This is especially critical in frail patients, who are more vulnerable to the infectious complications of prolonged TCE therapy and delayed neurotoxicity associated with CAR-T. For elderly frail individuals, finite-duration TCE therapy or CAR-T products with more favorable toxicity profiles may represent appropriate therapeutic strategies.

Indeed, among elderly patients (both frail and non-frail) for whom competing causes of non-myeloma mortality are common—and the need for subsequent lines of therapy therefore limited—the initial treatment choice is critical. The challenge ahead lies in individualizing therapy: selecting the right treatment for the right patient at the right time. This requires a nuanced approach that distinguishes between frail and non-frail individuals, balances efficacy with tolerability, avoids overtreating those with low-risk disease, and ensures high-risk patients are not undertreated.

To support this, prospective studies focused specifically on elderly and frail populations are urgently needed, including risk- and dose-adapted strategies to appropriately address the heterogeneity of this subpopulation.

## Figures and Tables

**Table 1:** Pivotal modern phase III clinical trials of triplet and quadruplet therapy in newly diagnosed transplant-ineligible multiple myeloma.

Trial, Treatment Combination	Sample Size, *n*=	Median Age Age ≥ 75 Years	HRCA *, %	ISS = III, %	ORR	CR	MRD-Negativity (10^−5^)	Median Follow-Up (Months)	Median Overall Survival (Months)	Median Progression-Free Survival (Months)	Treatment Discontinuation Due to AEs, %	Peripheral Neuropathy, % All Grades/Grade ≥ 3	Infections, % All Grades/Grade ≥ 3
**MAIA ^†^ [7,34]** **Rd**	369	74 44%	14	30	81	30	11	65	66	34	22	18/1	NA/29
**MAIA ^†^ [7,34]** **DRd**	368	73 43%	15	29	93	51	32	65	Not reached	62	13	28/2	NA/41
**ALCYONE ^±^ [32,33]** **VMP**	356	71 30%	15	36	74	24	6	40	Not reached	19	9	32/4	48/15
**ALCYONE ^±^ [32,33]** **D-VMP**	350	71 30%	17	41	91	43	22	40	Not reached	36	7	28/1	67/23
**SWOG777 ^‡^ [35,36]** **Rd**	225	63 NA	NA	35	79	12	NR	84	56 (>65 years)	24 (>65 years)	10	NA	31/16
**SWOG777 ^‡^ [35,36]** **VRd**	235	63 NA	NA	33	90	24	NR	84	65 (>65 years)	34 (>65 years)	23	NA	33/18
**CEPHEUS^¶^** **[37]** **VRd**	195	70 NA	14	28	93	62	39	59	Not reached	53	16	61/8	86/32
**CEPHEUS^¶^** **[37]** **D-VRd**	197	70 NA	13	28	97	81	61	59	Not reached	Not reached	8	56/8	92/40
**IMROZ ^^^ [38]** **VRd**	181	72 32%	19	NR R-ISS: 11	92	64	58	60	Not reached	45	26	61/6	87/38
**IMROZ ^^^ [38]** **Isa-VRd**	265	72 26%	15	NR R-ISS: 12	91	75	44	60	Not reached	Not reached	23	54/7	91/45
**BENEFIT ^+^ [39]** **Isa-Rd**	135	74 36%	NA	20	78	31	26	24	Not reached	Not reached	3	28/1	39/NA
**BENEFIT ^+^ [39]** **Isa-VRd**	135	73 31%	NA	16	85	58	53	24	Not reached	Not reached	4	52/3	47/NA

* HRCA = t (4;14), t (14;16), and Del17p. † DRd, 28-day cycles of IV daratumumab plus lenalidomide (25 mg on days 1–21 of each cycle) and dexamethasone (40 mg on days 1, 8, 15, and 22 of each cycle). ± D-VMP, 6-week cycles of subcutaneous bortezomib (1.3 mg/m^2^ on days 1, 4, 8, 11, 22, 25, 29, and 32 of cycle 1 and on days 1, 8, 22, and 29 of cycles 2–9), melphalan (9 mg/m^2^ once daily on days 1–4 of each cycle), and prednisone (60 mg/m^2^ once daily on days 1–4 of each cycle), IV daratumumab. ‡ No intent for immediate transplant, VRd, eight 21-day cycles of IV bortezomib (1.3 mg/m^2^ on days 1, 4, 8, and 11) combined with lenalidomide (25 mg on days 1–14 of each cycle) and dexamethasone (20 mg on days 1, 2, 4, 5, 8, 9, 11, and 12; VRd arm) or Rd alone (six 28-day cycles of lenalidomide 25 mg once a day on days 1–21 and dexamethasone 40 mg on days 1, 8, 15, and 22). On completion of induction, all patients received lenalidomide, 25 mg once a day for 21 days, plus dexamethasone 40 mg weekly of a 28-day cycle until disease progression. ¶ Eight 21-day cycles of VRd, consisting of subcutaneous bortezomib (1.3 mg/m2 on days 1, 4, 8, and 11), oral lenalidomide (25 mg on days 1–14), and dexamethasone (20 mg on days 1, 2, 4, 5, 8, 9, 11, and 12), then ongoing 28-day cycles of Rd, consisting of oral lenalidomide (25 mg on days 1–21) and oral dexamethasone (40 mg on days 1, 8, 15, and 22) until disease progression. ^ Four 6-week induction cycles followed by continuous Isa-Rd or Rd until disease progression. Induction consisted of subcutaneous bortezomib (1.3 mg/m^2^ on days 1, 4, 8, 11, 22, 25, 29, and 32), oral lenalidomide (25 mg on days 1 to 14 and 22 to 35), and dexamethasone (20 mg on days 1, 2, 4, 5, 8, 9, 11, 12, 15, 22, 23, 25, 26, 29, 30, 32, and 33). + All patients received isatuximab, lenalidomide, dexamethasone, and/or bortezomib from cycle 1 to cycle 12, followed by isatuximab + lenalidomide and/or bortezomib from cycle 13 to cycle 18, and isatuximab + lenalidomide from cycle 19 to progression. Lenalidomide was given orally at 25 mg on days 1–21 from cycle 1 up to progression. Dexamethasone was given orally at 20 mg weekly at days 1, 8, 15, and 22 until cycle 12, then was permanently stopped. Bortezomib was given at 1.3 mg/m2, weekly at days 1, 8, and 15, subcutaneously from cycles 1 to 12, and bimonthly at days 1 and 15 from cycle 13 to 18. NA, not available; R-ISS, revised-international staging system.

**Table 3:** Advantages, disadvantages, and future directions for FDA-approved CAR-T and T-cell engagers.

Characteristic	CAR-T	T-Cell Engagers
**FDA-approved agents**	Ide-cel Cilta-cel	Teclistamab Elranatamab Talquetamab
**Manufacturing time/availability**	4–8 weeks	**‘***Off-the-shelf*’ Immediate
**Treatment frequency**	‘*One-and-done*’, Once off therapy	Ongoing Q1–2 Weekly
**Hospitalization required**	Yes	Cycle 1 or not at all
**Specialized requirements**	Foundation for the accreditation of Cellular Therapy (FACT) or Joint Accreditation Committee ISCT-Europe & EBMT (JACIE)	Risk Evaluation and Mitigation Strategy (REMS) program
**Cytokine release syndrome** **All grades /≥ Grade3**	85–95/5	60–70/0–1
**ICANS, %** **All grades/ ≥ Grade3**	15/2	3–4/0
**Infections, %** **All grades/ ≥ Grade3**	58/20	BCMA: 70–75/40–45 GPRC5D: 76/20
**Hypogammaglobulinemia, %**	90	75–95
**Overall response rate, %**	75–95	60–70
**Median progression-free survival in late relapse (>3 prior lines)**	12–35 months	11–17 months
**Expense**	$$$	$$
**Future directions**	Maintenance strategies Streamlined manufacturing, reduce vein-to-vein time Improved bridging strategies Enhanced CAR-T design: armored CARs, dual antigen binding, etc.	Combination therapy Time-limited therapy Reduced intensity schedules Outpatient administration

**Table 4:** Ongoing phase III clinical trials of T-cell engagers or CAR-T in newly diagnosed transplant-ineligible multiple myeloma.

Trial, Treatment Combination	Sample Size	Population	Phase	Combination Therapies	Comparator Arm
**CARTITUDE-5 [116]**	Target *n* = 650	Transplant-ineligible or transplant not planned	3	Cilta-cel + Bortezomib Lenalidomide Dexamethasone	Bortezomib Lenalidomide Dexamethasone
**MAJESTEC-7 [117]**	Target *n* = 1000	Transplant-ineligible	3	Teclistamab + Daratumumab Lenalidomide Dexamethasone	Daratumumab Lenalidomide Dexamethasone
**MAGNESTISMM-6 [118]**	Target *n* = 646	Transplant-ineligible	3	Elranatamab + Daratumumab Lenalidomide Dexamethasone	Daratumumab Lenalidomide Dexamethasone
**Linvoseltamab** **NCT06932562**	Target *n* = 1000	Transplant-ineligible	3	Linvoseltamab + Daratumumab Lenalidomide Dexamethasone	Daratumumab Lenalidomide Dexamethasone
